# The Potential Use of Near Infrared Spectroscopy (NIRS) to Determine the Heavy Metals and the Percentage of Blends in Tea

**DOI:** 10.3390/foods13030450

**Published:** 2024-01-31

**Authors:** Isabel Revilla, Miriam Hernández Jiménez, Iván Martínez-Martín, Patricia Valderrama, Marta Rodríguez-Fernández, Ana M. Vivar-Quintana

**Affiliations:** 1 Food Technology, Universidad de Salamanca, E.P.S. de Zamora, Avenida Requejo 33, 49022 Zamora, Spain; irevilla@usal.es (I.R.); miriamhj@usal.es (M.H.J.); ivanm@usal.es (I.M.-M.);; 2 Department of Chemistry, Universidade Tecnológica Federal do Paraná (UTFPR), Via Rosalina Maria dos Santos 1233, Campo Mourão 87301-899, Paraná, Brazil

**Keywords:** red tea, black tea, green tea, NIRS (Near Infrared Spectroscopy), Residual Mean Square residuals

## Abstract

The following study analyzed the potential of Near Infrared Spectroscopy (NIRS) to predict the metal composition (Al, Pb, As, Hg and Cu) of tea and for establishing discriminant models for pure teas (green, red, and black) and their different blends. A total of 322 samples of pure black, red, and green teas and binary blends were analyzed. The results showed that pure red teas had the highest content of As and Pb, green teas were the only ones containing Hg, and black teas showed higher levels of Cu. NIRS allowed to predict the content of Al, Pb, As, Hg, and Cu with ratio performance deviation values > 3 for all of them. Additionally, it was possible to discriminate pure samples from their respective blends with an accuracy of 98.3% in calibration and 92.3% in validation. However, when the samples were discriminated according to the percentage of blending (>95%, 95–85%, 85–75%, or 75–50% of pure tea) 100% of the samples of 10 out of 12 groups were correctly classified in calibration, but only the groups with a level of pure tea of >95% showed 100% of the samples as being correctly classified as to validation.

## 1. Introduction

After water, tea is one of the most widely consumed non-alcoholic beverages in the world. Tea production worldwide had increased from 5.9 billion kg in 2018 to 6.8 billion kg in 2023 with a projected growth rate of 2.7%. The global tea market was valued at around 120.7 billion U.S. dollars in 2023 and is expected to grow annually by 5.3% until 2028 [1].

Tea is processed from the buds and new stems of *Camellia sinensis* which is native to southwest China [2]. The chemical composition of tea includes carbohydrates, amino acids, proteins, minerals, polyphenols, and alkaloids; it provides potential health benefits [3,4] and has important physiological properties [5]. Tea is considered to be antioxidant, anti-inflammatory, antiproliferative, antimutagenic, and antibacterial; it confers protection against cardiovascular diseases, hyperglycemia, metabolic disorders, and some cancers and may also reduce the risk of osteoporotic fractures in the elderly [6]. In addition to all of this, it contains caffeine, the consumption of which can provide a refreshing sensation, relieve pain and inflammation, encourage metabolism, and has diuretic effects [7]. 

Tea can be classified into six main categories based on their different processing techniques, which have been named green tea (unfermented), yellow and white teas (slightly fermented, 10–20%), Oolong tea (semi-fermented, 30–60%), black tea (fully fermented, 80–100%), and dark tea (post-fermented, ≤100%). In addition to these, Pu’er, also called red tea, is a unique microbial fermented tea produced from the sun-dried leaves of large-leaf tea species in the Yunnan province of China; it is attracting increasing interest which may be due to its multiple health benefits [8]. The composition, bioactive compound content, and antioxidant activity of tea are influenced by the different processing technologies applied, which means that different types of tea obtained from the same cultivar may vary greatly [9]. Green tea has thus been shown to provide protection against cancer by scavenging free radicals, dark tea may decrease the risk of cardiovascular disease, and black and oolong teas seem to possess outstanding antioxidant properties [10].

Based on their mineral composition, the leaves of *Camellia sinensis* are a source of elements such as zinc, manganese, iron, copper, magnesium, titanium, aluminum, strontium, bromine, sodium, potassium, phosphorus, iodine, and fluorine. Some of these minerals such as Fe, Mn, and Zn are components of important enzymes or are involved in a number of physiological processes, which means they are considered essential [11,12]. However, some elements are toxic to human health, such as As, Cr, Cd, Co, Ni, and Pb [13,14].

Moreover, different growing areas show variations in growing conditions, which include climate, rainfall, altitude, soil, fertilizers, microelements, and processing procedures [15], and these are all factors which contribute to the chemical composition of tea leaves [16]. The mineral composition of tea leaves appears to be influenced by the geographical origin, soil type, tea variety, climatic conditions, and other seasonal changes [17]. The presence of toxic heavy metals such as mercury, lead, and cadmium in tea leaves is probably related to soil contamination, atmospheric deposition, chemical and organic fertilizer applications, or irrigation with contaminated water and waste disposal [6]. High levels of heavy metals in the soil could inhibit the growth of tea trees by altering the biomass while reducing tea quality by changing the content of major ingredients [18] and therefore the quality of the tea. For instance, the application of phosphate fertilizers may be a major source of Cd in agricultural soils [19,20]. On the other hand, inorganic amendments were mainly responsible for the increase of Cr and Cu in tea growing soils while organic amendments were the influential factors in the enrichment of other metals [19]. 

As a result, the determination of mineral composition in tea leaves is relevant to industry and consumers. It is usually carried out by means of atomic absorption spectrometry (AAS) [12,21,22,23], which has low operational costs and provides good analytical performance although only a few elements can be determined simultaneously [24] by inductively coupled plasma mass spectroscopy (ICP-MS) or inductively coupled plasma atomic emission techniques (ICP-AES) which are accurate and expensive [25,26,27]. X-ray fluorescence (XRF) spectroscopy is an emerging screening technique for determining the mineral/elemental content in teas [4,11]. It is fast and accurate, non-destructive, and requires only minimal sample preparation. One of its limitations is the partial sensitivity of the method for high mass elements, which is due to high X-ray scattering (which can result in increased background intensities) [24]. 

In contrast, near infrared spectroscopy (NIRS) is a rapid, simple, multi-parametrical, and non-destructive technique. Moreover, NIRS shows excellent analytical performance in the prediction of the main contents of tea, including tea polyphenols, catechins, caffeine, theaflavins, chlorophyll, and sensory properties [7,28,29]. However, although NIR spectroscopy has been successfully used to predict the mineral composition in several matrices such as meat, cheese, pulses, quinoa, alfalfa, and propolis [30,31,32,33,34,35], as far as we know it has not been used for this purpose in tea.

Blending of teas with different characteristics is a common practice in the tea industry for several reasons, such as reducing costs by using lower grade teas [36], improving quality by obtaining a better taste [37] and fulfilling the consumer expectations by preparing teas with new characteristics or standardizing the sensory properties of a brand [36]. Moreover, blending different herbal teas of different origins and characteristics with tea to take advantage of the specific sensory properties and some medicinal values of this plants is increasingly consumed as wellness beverages [38,39]. In fact, a wide range of blends has been studied, from very low [40] to high blending percentages, up to 50% in some case. These blends can be made from different types of tea or by mixing tea with other plants for the preparation of other herbal teas [38,41,42]. In this scenario, product quality control and fraud prevention by operators is becoming increasingly important. In fact, fraud control is mandatory for industries certified according to GFSI (Global Food Safety Initiative) standards, such as IFS Food (International Featured Standards) and BRCGS Food (British Retail Consortium Global Standards). For example, mixing with leaves of lower quality species is considered adulteration [43]. Therefore, nowadays clean, low-cost and non-invasive techniques have been tested to assess the mixture, such as hyperspectral imaging [40], electronic nose [41], or visible and near-infrared spectroscopy [42,44]. 

Based on this background, the main purpose of this work was therefore to study the potential of near infrared spectroscopy for predicting the metal composition (Al, Pb, As, Hg and Cu) of green, red, and black tea samples and their mixes. In addition, the possibility of establishing classification models for the three types of tea (green, red, and black) and their blends using mineral composition and NIR spectra was also considered. 

## 2. Materials and Methods

### 2.1. Samples

A total of 322 samples were analyzed. Samples of commercial black (7 samples), green (11 samples), and red (8 samples) teas were purchased in local markets (Zamora and Salamanca, Spain). The binary blends of two out three tea types were prepared in variable percentages ranging from 99 to 50% of tea of interest (1 to 50% of second tea) and thoroughly mixed. As the samples were purchased in supermarkets or small shops data on the geographical origin, the processing conditions, and the season they were picked were not available. The 16 pure samples, corresponding to 7 black, 11 green and 8 red tea samples, were analysed for their mineral content. In addition, the 65 samples that were used for the external calibration of the model were also analysed, these samples consisted of randomly selected mixed samples in different proportions. For the remaining samples (241) the metal contents were calculated based on the metal concentrations of each of the samples used to make each mixture 

To ensure that the best mixing of the samples was obtained, a mixing protocol was established. The protocol conditions were as follows: the individual amounts of each of the teas used were weighed and placed in a hand-operated drum mixer with 500 g capacity containers for 15 s. Both the mixtures (241) for which the metal content was calculated and the 65 mixtures for which the metal content was established by laboratory analysis were prepared following this protocol.

### 2.2. Chemical Analyses

Element concentrations of Al, As, Cu, Hg, and Pb were determined by ICP-MS in pure teas (7 black, 11 green, and 8 red teas) and in the 65 samples of the external validation test. Approximately 0.2 g of each sample was placed in Teflon vessels with 5 mL of HNO_3_ and then placed in a Milestone microwave digestion system (Ethos Sel Milestone, Ontario, ON, Canada) and 1000 watts of power was applied for 5 min. The samples were allowed to cool and then 5 mL of HNO_3_ and 1 mL of H_2_O_2_ (30%) were added. The samples were then treated with 1000 watts for 10 min. When the samples were at room temperature, they were made up to 100 mL with distilled water and stored at 4 °C until analysis. The concentrations of the analyzed elements were quantified using an Agilent 7800 ICP mass spectrometer (Agilent, Santa Clara, CA, USA). The working conditions were as follows: reference power 1550 W, plasma airflow 15 L/min, auxiliary airflow 0.9 L/min, nebulizer airflow 0.99 L/min. Certified standard solutions of 1g/L concentration (Panreac, Barcelona, Spain) were used, grouping the elements into two multi-elemental standards. The results were expressed in mg kg^−1^ of sample for As, Cu, Hg and Pb and in g kg^−1^ for Al.

### 2.3. Near Infrared Spectroscopy

A total of 10 g of each of 322 samples (7 black, 11 green, and 8 red teas and the binary mixtures of black and red, black and green, and green and red tea prepared ranging from 50 to 99% of the tea of interest) were analyzed using the Foss NIRSystem 500 (Hillerod, Denmark) with the transport half-cup which has a 11.6 × 3.7 cm window. Spectra were recorded from 1000–2498 nm every 2 nm and 32 spectra were performed at each recording. Three spectra of each sample were recorded and then the average spectrum was calculated. The diffuse reflectance signal of the NIR spectrum was expressed as log (1/R) (R = reflectance). The software used for data manipulation and chemometric analysis was Win ISI 4.10. In order to develop the models 80% of the samples (257) were taken in the calibration group and 20% of the samples (65) in that of external validation, the samples were place in the two groups at random.

### 2.4. Chemometric Techniques

The metal content was quantified by the modified partial least squares (MPLS) regression method [45]. In MPLS the NIR residuals at each wavelength were obtained after each factor had been calculated and standardized (by dividing the standard deviations of the residuals at each wavelength) before calculating the next factor. To minimize the undesired contributions to the NIR signals, various spectral pretreatments were used together with derivatives and smoothing. The effects of scattering were removed using multiplicative scatter correction (MSC), standard normal variate (SNV), detrend (DT), or SNV-DT and combinations of these [28,33,46]. Moreover, several mathematical treatments were tested in the development of the NIRS calibrations where each of one was coded with four digits (2,4,4,1, for example), in which the first digit is the number of the derivative, the second is the gap over which the derivative is calculated, the third is the number of data points in a running average or smoothing, and the fourth is the second smoothing [33]. The number of variables was reduced by principal component analysis (PCA). A sample with an H statistic and ≥3.0 standardized units from the mean spectrum was defined as a global H outlier and eliminated from the calibration set. The calibration process was implemented with the spectra of the resulting samples and their chemical data. Using the criterion T ≥ 2.5, samples with high residual values when predicted were removed from the set. The statistical parameters of the calibration were obtained for each of the components after removing the samples for spectral (H criterion) or chemical (T criterion) reasons. To avoid overfitting during the development of the equations using MPLS, it is recommended to apply a cross-validation to select the optimal number of factors [47]. Therefore, the calibration set is divided into groups, in this case four due to the number of samples. Then, each group is validated using a calibration developed on the other samples. Finally, validation errors are combined into a standard error of cross-validation (SECV) [48]. The SECV has been reported to be the single best estimate of the predictive ability of the equation. This statistic is similar to the mean standard error of prediction (SEP) of ten randomly chosen prediction sets [49]. The statistics R squared (RSQ, multiple correlation coefficients) and SECV were used to select the best equations.

### 2.5. Pattern Recognition

Unsupervised pattern recognition by means of Principal Component Analysis (PCA) was performed to highlight the heavy metals which could be markers to discriminate the different teas (green, black, or red). The calculations were performed using the heavy metal results of the pure samples through the MATLAB software R2007B (The MathWorks Inc., Natick, MA, USA) and PLS-toolbox 5.2.

### 2.6. Discriminant Analysis 

The samples were discriminated according to their purity by using different approaches and methods. The first approach was to study the feasibility of discriminating each type of the pure samples (black, red, and green) from each type of the following blends: black blends containing more than 50% of black tea; red blends containing more than 50% of red tea; and green blends containing more than 50% of green tea, which means that six groups were analyzed. The second approach was to study the feasibility of discriminating the percentage of the blend. To do so, four categories of each type of tea were considered: blends containing more than 95%, blends containing between 95% and 85%, blends containing between 85% and 75%, and blends containing between 75% and 50% of the selected tea (black, red, or green). Twelve categories were therefore considered in this approach

The SIMCA method (soft independent modelling of class analogy) was used to classify the samples using the whole NIR spectrum. The data (700 pieces of data corresponding to the spectrum from 1100 to 2498 nm, each measuring 2 nm) were normalized, scaled, and mean-centered for SIMCA modelling. First of all, a PCA was applied, and a new set of variables were calculated by linearly transforming the original variables into a set of new components (PCs) which retain the information of the original set. The number of PCs for classifying purposes was selected using the criteria of having an eigenvalue of >1. The projection of the samples in the space determined by the first PCs allowed the detection of groups present in the samples. Afterwards, the OPLS-DA (orthogonal projection latent structure discriminant analysis) method was carried out on the training set (80% of the data) and on the validation set (20% of the samples) in order to test the robustness of the discriminant model. The software used was SIMCA-P software version 14.1 (Umetrics, Sartorius Stedim Biotech AS, Umeå, Sweden).

The RMS-X residual (residual mean squares) method was also used. In this method the square root of the arithmetical mean of the residual mean squares (RMS or the quadratic mean) is calculated to obtain the RMS-X residual values. Different mathematical treatments, derivatives, and smoothing procedures coded as previously reported (i.e., Detrend (2,4,4,1)) were tested both for the calibration set (80% of the samples) and for the validation set (20% of the samples). The sensitivity (the proportion of samples belonging to one specific group which are correctly identified) and selectivity (the proportion of samples not belonging to that group which are correctly identified) were also calculated using the expressions proposed by [50]. The combination giving the highest percentage of correctly classified samples together with the higher selectivity and specificity was selected as the most suitable. The WinISI 4.10 software was used for this analysis.

## 3. Results and Discussion

### 3.1. The Heavy Metal Content of the Samples

The contents of the five heavy metals quantified by ICP-MS in pure samples of green, black, and red tea are shown in Table 1. Statistical analysis showed significant differences between samples for all of the heavy metals. Black tea samples showed significantly lower contents of Al, Pb, and As, while green tea showed the lowest values of cupper and was the only type of sample to show the presence of Hg. On the other hand, red tea showed the highest values of As and Pb.

The mean values observed for aluminum are above the range previously reported for black 0.4–1.2 g/kg [27,51,52,53], green 0.5–1.41 g/kg [53,54,55], and red tea 1.44 g/kg [26]. In accordance with our results, these studies highlight the fact that green and red tea showed higher values of aluminum than black tea [26,53]. As regards Pb, our results are fall within the ranges previously reported in the literature for black tea (0.15 to 2.31 mg/kg), green tea (0.10 to 1.93 mg/kg), and red tea (0.10 to 4.66 mg/kg) [9,21,55,56,57,58,59,60,61]. Moreover, the highest amounts of Pb were observed in red tea followed by green and black tea as reported in the aforementioned studies and others comparing the three types of tea [59]. Less research has been carried out on the arsenic and mercury content. The reported values for As varied between 0.09 and 0.39 mg/kg in black tea [57,62,63,64], between 0.038 and 0.088 mg/kg in green tea [55], and between 0.07 and 0.25 in red tea [9,60]. The levels of this heavy metal shown in Table 1 were within the range described for red tea and in the lower part of the range for black and green tea. As far as Hg is concerned, the reported intervals are 0.04–0.61, 0.032–0.22, and 0.01–0.03 mg/kg for black, green and red tea, respectively, [55,60,62,65]; the results found in the teas commercialized in Spain were therefore lower than previously reported. Finally, the results reported for copper show a high variability ranging from 11.29 to 44.38 mg/kg and from 4.7 to 36.5 mg/kg for black [27,57] and green tea [58], respectively. However, the most frequent values are between 10 and 26.5 mg/kg for both types of tea [21,27,53,55,57,61,62]. On the other hand, the reported values for red tea showed less variability and are within this range (14.34–21 mg/kg) [9,26,60,66,67] as was observed in the results obtained during this study. The current results also agreed with the studies of Gabrowska et al. [65], Konieczynsky et al. [67] Mackenzi et al. [26], and Czernicka et al. [66] who found that black tea showed higher levels of copper than green tea, while red tea tended to show similar values to black tea [26,65].

### 3.2. Exploratory Analysis of the Samples according to Their Heavy Metal Content

**Figure 1:**
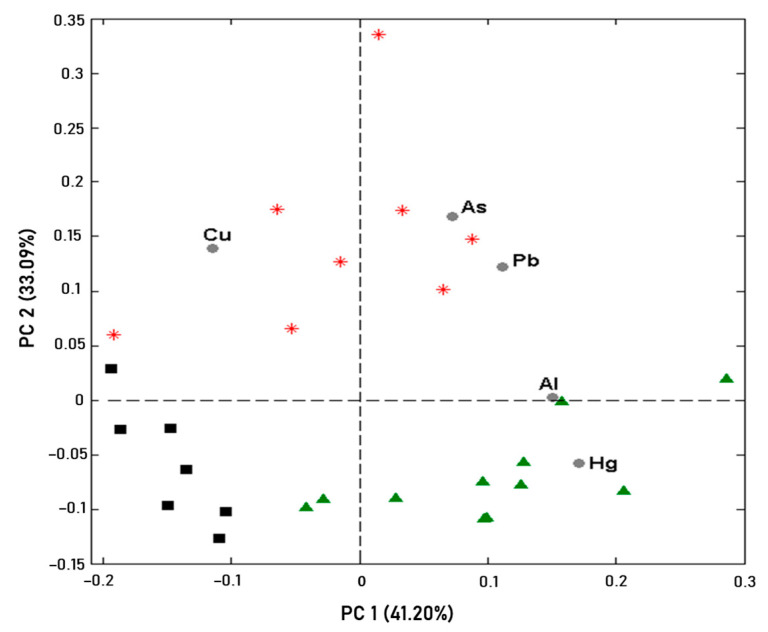
PCA results. (■) black (∗) red. (▲) green tea pure samples.

PCA was applied by means of the autoscale preprocess to extract more information from the heavy metal results obtained in the pure samples. Two major components (PCs) reflected 74.29% of the variance explained. The projection of the samples and the variables on the two first PC’s is shown in Figure 1.

PC2 reveals a distinction for the red tea samples on the positive side. As far as the loadings are concerned, samples of green tea are grouped by the Al and Hg results, while red tea is separated based on the Cu, As, and Pb results. In general, green tea presents higher concentrations of Al and Hg compared with red and black tea. On the other hand, the red tea samples showed higher concentrations of Cu, As, and Pb than green and black tea. The black tea samples presented a lower concentration of heavy metals. These results therefore indicated that it was possible to classify the tea types according to their heavy metal content.

### 3.3. Determination of the Heavy Metal Content Using near Infrared Spectroscopy

#### 3.3.1. Spectra of the Samples

**Figure 2:**
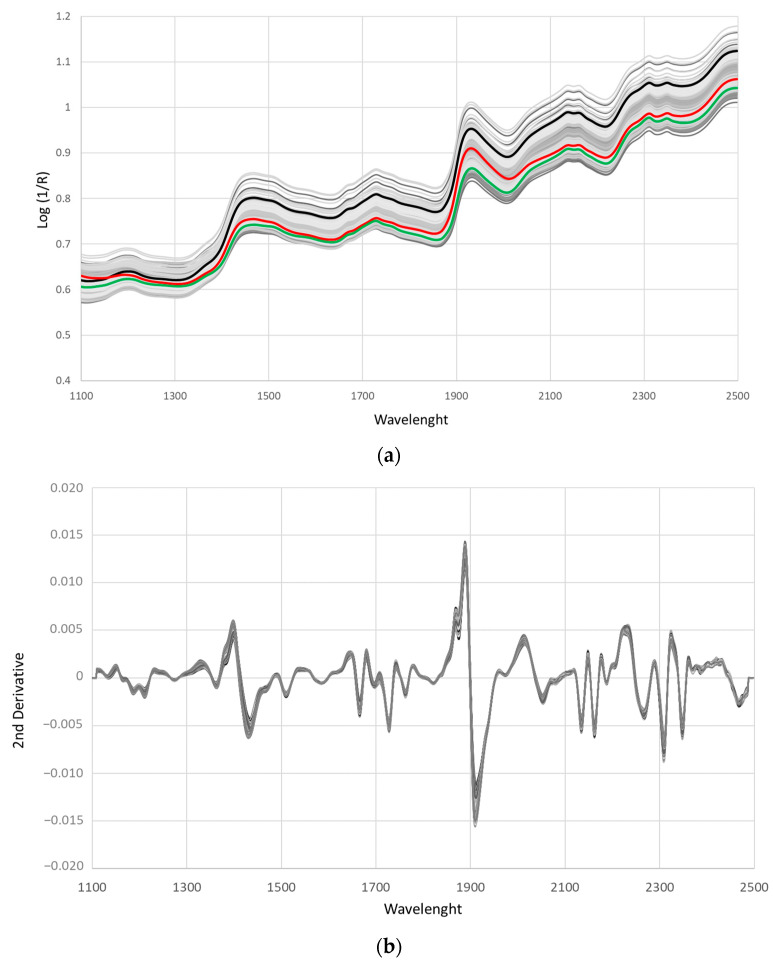
(**a**) Raw spectra of the 322 samples and mean NIR spectra of the black (**-**), red (**-**) and green (**-**) tea pure samples and (**b**) spectra after second derivative Detrend 2,4,4,1.

In order to perform the calibration equations, binary mixtures of black and red, black and green, and green and red tea were prepared ranging from 1% to 50% of each pair. The spectra of the pure teas (7 black, 11 green, and 8 red teas) and the mixtures were measured using the transport device of Foss NIRSystem 5000 (DK-3400, Hillerød, Denmark), equipped with the transport half-cup which has a 11.6 × 3.7 window. Figure 2a shows the spectra of the 322 samples directly obtained from the equipment, together with the average spectrum of the pure samples of each tea type, and Figure 2b the spectra after the pre-treatment Detrend (2,4,4,1) which gave the best calibration and validation results as was subsequently observed.

The main absorption peaks were found at 1440 and 1950 nm corresponding to O-H stretching and O-H deformation; information was therefore provided on the water content of the samples. According to the spectra, these peaks were larger for black tea followed by red and green tea. The peak founds at 1220 and 1670 nm corresponded to the C-H stretch second overtone and the C-H first overtone, while the peak found at 1740 corresponded to the -CH_2_ overtone. These bands have been linked to the presence of ingredients such as catechins, tea polyphenol, and alkaloids [68,69,70,71]. The region between 2210 and 2500 nm included a combination of C-H stretching and C-H deformation and a 2nd overtone of C-H deformation. This spectral region was linked to the presence of polyphenols, catechins, and teaflavins [72] but also to that of cellulose and caffeine [28,73]. Other peaks observed in the spectra were found at 2150 and 2170 nm and associated with NH_2_ vibrations in amide I groups + NH bending in amide III groups, which possibly corresponded to free amino acids and proteins [74]. These spectral features were even more clearly observed when a second derivative was applied (Figure 2b). In addition to the region between 2210 and 2500 nm or the region between 2100 and 2200 nm which showed a large number of peaks related to the phenolic and protein composition of the samples, respectively, already described, another spectral interval which showed a high number of peaks was the region between 1600 and 1800 nm corresponding to the CH-first overtone and the S-H first overtone of both total catechins and caffeine [60]. The peaks found between 1870 and 1900 corresponding to the C=O stretch overtone of TF2A or to the O-H combination and the C-H first overtone of TF2B are noteworthy [75]. Other peaks which can be observed more clearly in the second derivative are those at 1330 and 1400 nm which could be attributed to the first overtone of the C-H combinations of free amino acids [72].

The mean spectra of pure samples were similar to those previously described for black [46,69] green [74,76], and red teas [60] (Figure 2a). Black tea showed higher absorbance in all of the regions of the spectrum, while although the red and green tea spectrum showed similar absorbance, red tea always presented higher absorbance, especially in the band located at 1940 nm. This result suggests a higher water content in black tea followed by red and green tea. According to previous results the water content of different types of tea is highly variable and while some reports found a higher water content in black than in green teas [66], other studies reported the opposite [65] or similar values of water for black and green [77] or for black and red teas [65]. On the other hand, in general black teas showed lower levels of catechins and tannins and a higher caffeine content [65,67,77] because as the fermentation progresses the catechin content decreases and the caffeine increases [78]. As far as the protein content is concerned, it has been reported that it is very low in Purer tea [78] while the values for black and green tea showed a strong variability. In some cases, the values were similar for black and green tea and in others they were higher in green than in black tea [77,78,79].

Based on the mineral composition, a relationship between a particular absorption band and a particular mineral content cannot be established. This is due to the fact that there is no direct relationship between the mineral and the spectrum. Therefore, its determination is possible thanks to the associations of the minerals with other compounds present (this factor being different for each food). 

#### 3.3.2. Calibration Equations

The calibration was carried out by using the modified partial least squares regression (MPLS) method. To do so, the first step was to perform the principal component analysis (PCA). The results of the PCA showed that for all of the minerals using ten principal components the spectral variability explained was higher than 97%. In order to remove the samples which did not fulfil the spectral requirements, the Mahalanobis distance (H distance) was applied so that those samples with H values equal to or higher than 3 (1 sample) were removed from the sample set. Subsequently a calibration set including 80% of the samples (257) and a validation set with 20% of the samples (65) was built in which the mean, maximum values, and minimum values were similar for both sets (Table 2).

Table 3 shows the statistical descriptors of the best calibration equation obtained for each of the mineral elements (Al, As, Cu, Hg, and Pb). The best equation was determined according to RSQ, SEC, SECV, and RPD values. The final number of samples used for calibrations was the result of applying the T criterion. The samples with a T value exceeding 2.5 therefore had to be removed from the set because they were different from the population for chemical reasons. This number of samples depended on the component calibrated and ranged from 12 to 21. The calibration margins (the estimated minimum, the estimated maximum) were similar to or even broader than the range of values determined in the samples.

It can be observed that according to the results obtained with an RSQ of between 0.89 and 0.97 it is possible to predict all of the heavy metals in teas of varying compositions without any previous treatment or handling.

#### 3.3.3. Validation

**Figure 3:**
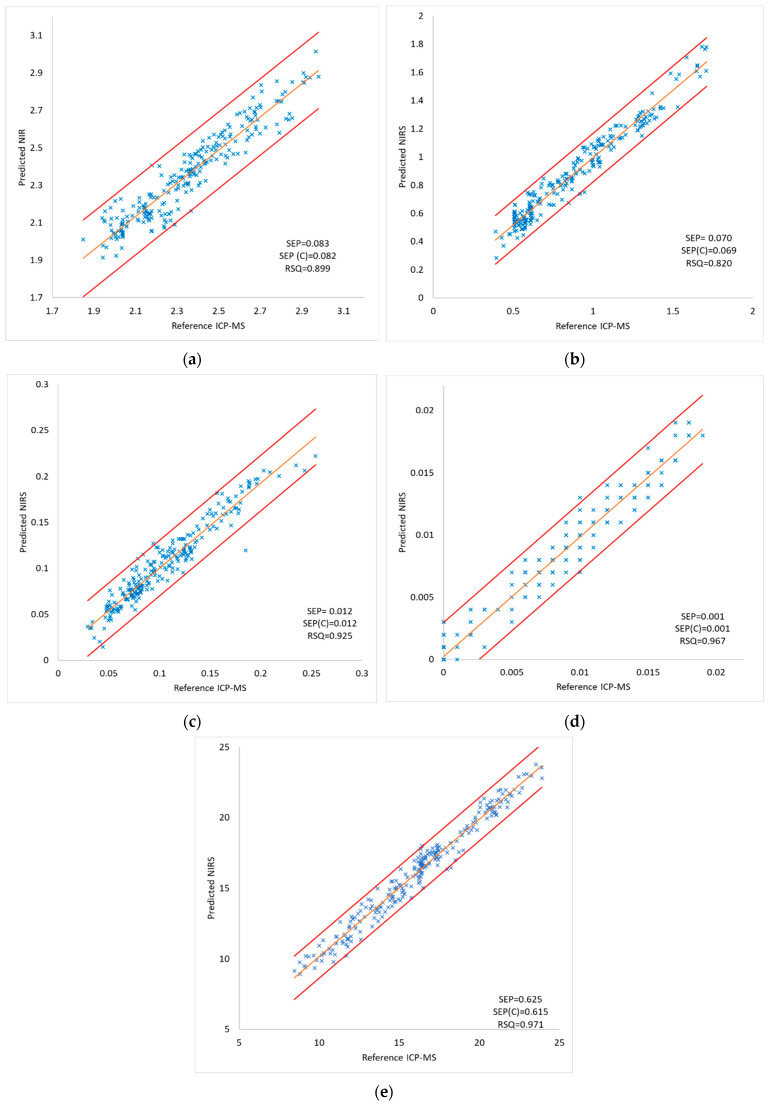
Comparison between the reference values determined by ICP-MS and the predicted values for the internal validation set (n = 257) for (**a**) aluminium, (**b**) lead, (**c**) arsenic, (**d**) mercury, (**e**) copper. SEP: standard error of prediction; SEP(C): standard error corrected by the bias; RSQ: multiple correlation coefficient.

The internal validation of the model was carried out by means of the cross-validation method. In order to do so, the calibration set was divided into four cross-validation groups, of which three were taken for the calibration set and one for the prediction set. This process was repeated four times in order to allow all of the groups to pass through a calibration and a prediction set. With this step the prediction capacity of the model was checked. Figure 3 shows the correlation of the reference values (obtained by ICP-MS) and the predicted values using NIR spectra with RSQ values ranging from 0.820 for aluminum to 0.971 for copper. 

The prediction capacity of the model was evaluated with the RPD parameter; a value greater than 2.5 indicated that the model can be considered adequate. All of the RPD values were above 2.5, ranging from 3.11 for aluminum to 5.78 for copper, which reveals that the prediction capacity of the model was suitable for predicting heavy metal contents in unknown samples of tea.

**Figure 4:**
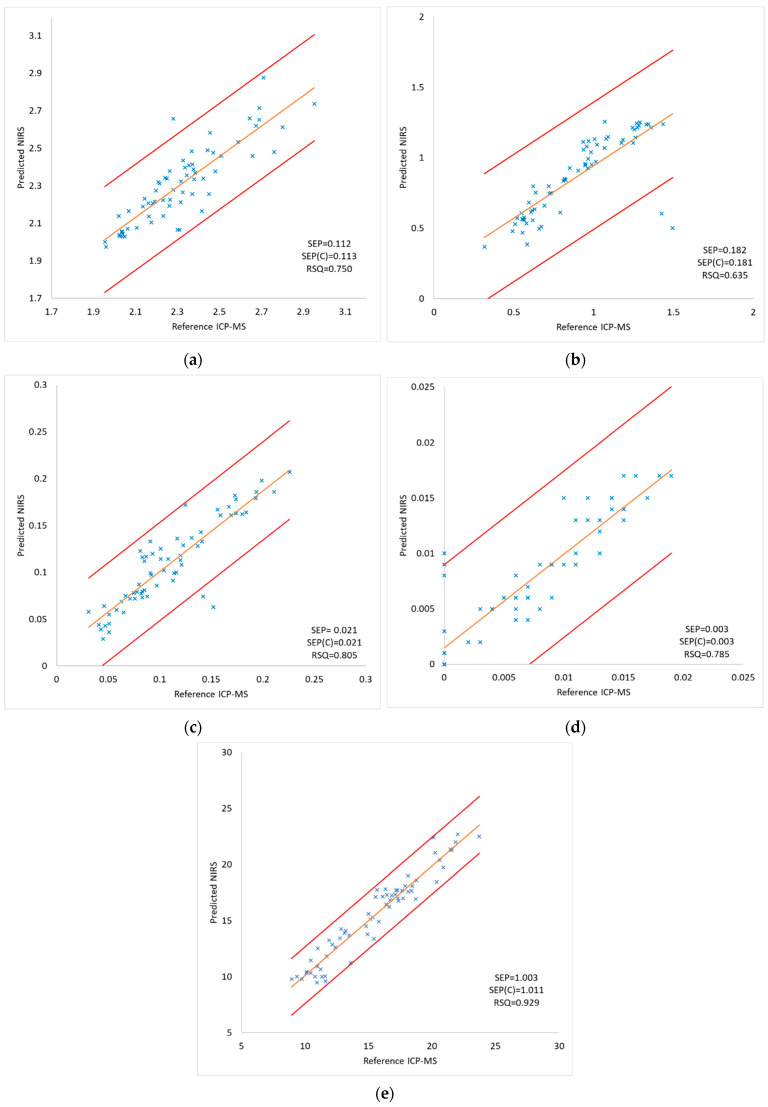
Comparison between the reference values determined by ICP-MS and the predicted values for the external validation set (n = 65) for (**a**) aluminium, (**b**) lead, (**c**) arsenic, (**d**) mercury, and (**e**) copper. SEP: standard error of prediction; SEP(C): standard error corrected by the bias; RSQ: multiple correlation coefficient.

To check the robustness of the equations we applied them to the NIR spectra of the samples included in the external validation set (n = 65), the heavy metal composition of which was representative of the whole sample set (Table 2). Figure 4 shows the comparison between the values obtained after applying the equations and the reference values obtained by ICP-MS. The RSQ values ranged from 0.635 for lead to 0.929 for copper while the SEP and SEP(C) values were very similar for all of the heavy metals analyzed. The values obtained by both methodologies were compared using Student’s test-values for paired values. The p values (Table 4) were higher than 0.05 for all of the components; it should be pointed out that there were no differences between reference and predicted values. It can therefore be concluded that both analytical methods give similar results. Table 4 also shows the RMSE (root mean standard error) values which were low for all of the heavy metals.

These results are in accordance with those of previous studies which indicated that it was possible to predict the mineral content of different vegetable matrices with good RSQ reaching values of 0.98 for K and Na for example in alfalfa and flour, respectively, [30,80]. However, for the microelements which are found in smaller quantities in the samples, the RSQ values attained lower values ranging from 0.41 to 0.64 for Mg, 0.64 to 0.95 for Fe, and 0.41 to 0.84 for Mn in products such as alfalfa, rocket (*Eruca versicaria*), artichoke, quinoa, *Curcubita pepo*, and *Brachiaria* [31,34,81,82,83,84]. Data on the calibration of heavy metals with NIRS are scarce; it has been reported that copper can be predicted with RSQ values ranging from 0.52 to 0.76 [81,85], while RSQ values of between 0.70 and 0.79 have been reported for aluminum and lead [86]. NIR spectroscopy can detect macronutrients such as N, P, and S because they are major constituents of NIR-sensitive organic compounds, whereas micronutrients and macronutrients which exist mostly in inorganic forms such as Ca, Mg, and K are detected by means of association with organic compounds and indirect correlation with organic compounds [87]. Therefore, there is not a direct absorption peak of metal components in the NIR region [88].

However, according to the general lineal model, the predicted concentration of a mineral (Y) can be explained by the next equation Y = β_0_ + β_1_x_λ1_ + β_2_x_λ2_ + β_3_x_λ3_ +……+ β_n_x_λn_, in which β are the coefficients measuring the contribution of each wavelength and x_λ1_, x_λ2_, x_λ3_, ………. x_λn_, are the wavelengths. Therefore, the β coefficients can help to correlate the metal content with specific wavelengths which in turn are correlated with the bonds of different functional groups because the highest the β value the highest the contribution of this wavelength.

**Figure 5:**
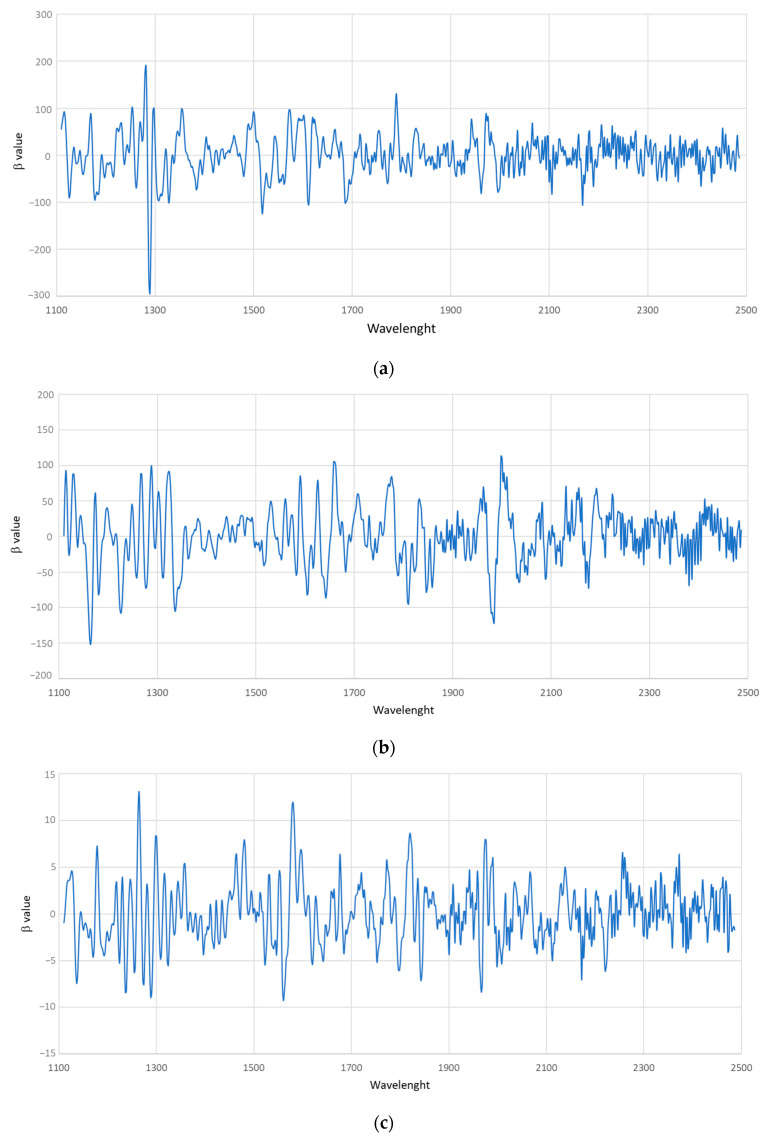

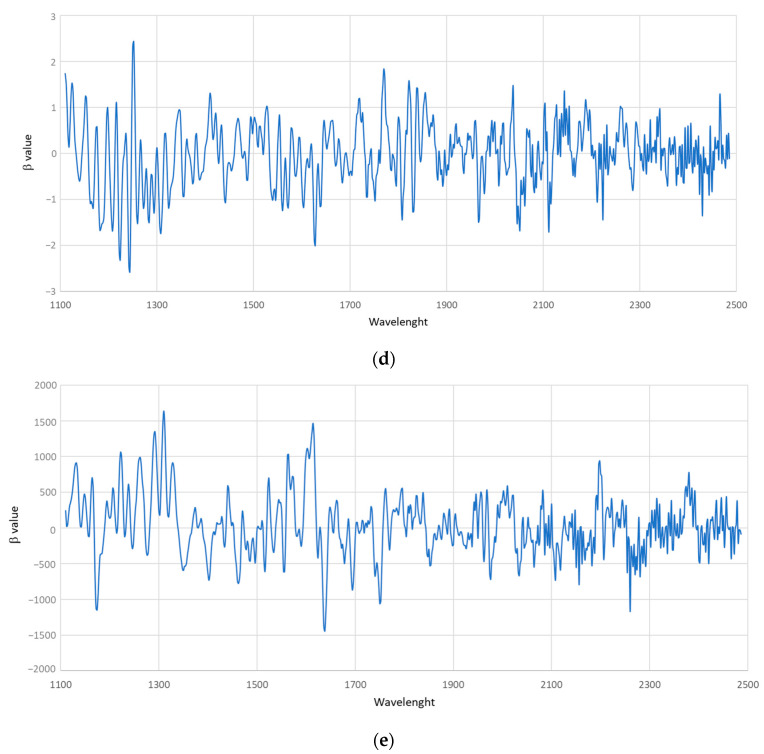
Values of the β coefficients for the calibration (**a**) aluminium, (**b**) lead, (**c**) arsenic, (**d**) mercury, and (**e**) copper.

Figure 5 shows the β coefficients corresponding to the calibration equations of the five heavy metals calibrated. It can be observed that Al had two large peaks at 1282 and 1290 nm, the two highest peaks for Pb were found at 1164 and 1984 nm, As showed the highest values for β coefficients at 1264 and 1580 nm, Hg at 1244 and 1252 and finally Cu at 1310 and 1614 nm. Besides this, Figure 5 allows to observe that all of the peaks with the highest values, both negatives and positives, for each of the calibrated metals were in the spectral regions between 1110 and 1300 nm, in the region between 1500 and 1700 nm pointing out that these regions had a large influence on the developed models. The first one (1110–1300 nm), which corresponds with the C-H second overtone, can be attributed to the presence of the catechins, tea polyphenols and the TF2B theaflavin, while the region between 1500 and 1700 correspond to the O-H and N-H bond and can be related with the presence of catechins and caffeine. Other spectral regions that showed high values of β coefficients were sited at 1800 which could be due to C=O bond stretch second overtone of the TF2A theaflavin and O-H combination and C-H first overtone of the TF2B [72]. There was, therefore, a strong correlation between phenolic compounds and metal content. This was because phenolic compounds, especially flavonoids, tend to form stable complexes with metals such as iron, chromium, nickel, copper, or lead [89,90]. 

Previous work has shown high correlations between trace minerals and the spectral region 1100–1300 in forages and legumes as previously reported while [33] found that trace mineral concentration in lentils showed a strong correlation with absorbance in the range 1510–1550 nm. A significant correlation between minerals and the absorption bands near 1400 nm and the spectral region between 1600 and 1700 has also been described for the calibration of minerals in grape derivatives [85,88] as observed in tea. Similar results, although with differences in the specific wavelength where the absorption maximum appears, have been described for other vegetables such as rocket, pumpkin fruits or flours [80,81,82]. This is due to the fact that mineral trace elements are bound to complexes and chelates that appear to be different among the plant matrix.

Regarding the metals calibrated in this work, Al content in propolis has been correlated with absorption peaks at 1336 and 1556 nm while Cu showed correlation with β coefficient peaks at 1244 and 1356 nm [32], as were observed in this work for tea, which were attributed to phenolic content. In addition, [87] also found absorption peaks in the 2200–2400 range that were attributed to the affinity of copper for proteins and chloroplasts. With regard to Pb, this heavy metal showed two important peaks in tea, the previously mentioned at 1984 and another at 2000 nm in agreement with the values reported in propolis which were 1820 and 1968 [32] although another study showed that the bands at 548 and 718 nm were most likely related to the lead content in tea [91]. Arsenic levels also appear to be correlated with bands in the visible part of the spectrum (674 nm) and with the bands at 1916, 1956 and 1966 nm observed in prostrate amaranth [92]. These authors considered that as absorption affected cellular absorbers such as chlorophyll and other macromolecules such as proteins and lipids. These results are in agreement with the peak observed in Figure 5 at 1966 nm for As. The data related to mercury are very sparse and indicate that the wavelengths at 1011, 1173 and 1295 nm, which are in the same range reported in this work for tea, were particularly important for the estimation of Hg in soils [93]. Finally, it is noteworthy that Al, Pb, As and Cu showed a peak at 1290 nm with a high and positive value for Al and Cu but with a negative value for Pb and As. This opposite behavior for the same wavelength depending on the mineral was also found by Cozzolino et al. [88].

### 3.4. Discrimination of the Percentage of the Blends Using near Infrared Spectroscopy

The spectra of the pure tea samples and the different binary mixtures were analyzed in order to study the feasibility of discriminating each type of the pure samples (black, red and green teas) from their blends and the feasibility of discriminating the percentage of the blend for each type of tea. Two methods were assayed (SIMCA and RMS-X residuals) and for each of them the total of the samples was divided into a training set with 80% of the samples while the remaining 20% of the samples built the validation set.

The analysis of the whole spectra with the SIMCA method for discriminating the pure samples vs. their blends involved a PCA analysis of the samples which gave a result of three PCs with an eigenvalue of >1 which explained the 99.6% of the total variance. The representation of the samples in the space defined by the three PCs did not show a good separation between the six groups. Subsequently a classification by the OPLS-DA method was carried out and the results are shown in Table 5. It can be seen that 100% of the pure black samples were correctly classified for both the calibration and validation sets and 98% and 93% (calibration and validation) of the pure red samples were correctly classified. However, only 45% of the pure green samples were assigned to the correct class in the calibration step and this value fell to 10% for the validation group while green blends were better classified (77% and 75% in the calibration and validation groups). On the other hand, only 25% of the classifications were correct for the red blends in both sets while 0% of the black blends were correctly classified.

**Figure 6:**
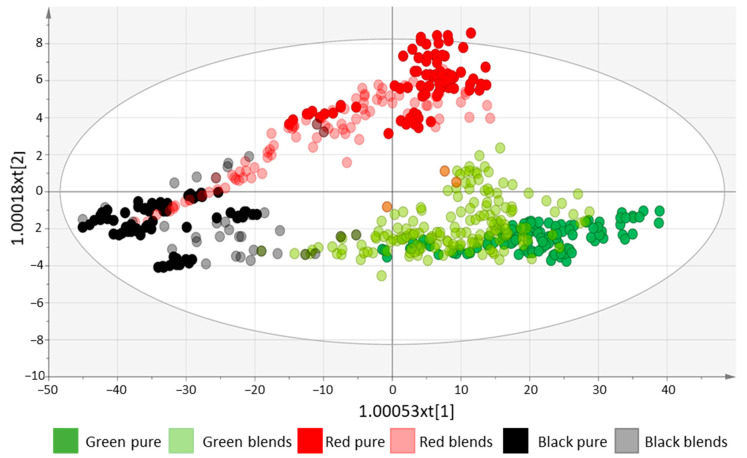
The OPLS-DA score plot of the black, red and green pure teas and the black, red and green blends (blends with more than 50% of each tea type) (two predictive and two orthogonal components, R2X = 0.569, R2 = 0.314, Q2 = 0.318).

The OPLS-DA score plot of the six groups (Figure 6) showed that pure red, green, and black samples were well separated, but the blends (mainly black and red blends and green and red blends) appeared distributed along the axes connecting the pure samples.

The results of the OPLS-DA for the groups which differed as to the tea type (black, red, and green) and in the percentage of blending (>95%, 95–85%, 85–75% or 75–50%) are shown in Table 5. Once again, the discrimination results for pure or very high percentages of each tea sample were good. Therefore, 100% of the samples containing >95% of the black or green teas were correctly classified for both the calibration and validation sets, while for red teas the values were 91% and 88%, respectively. However, the model was not able to classify correctly any of the samples of the remainder of the blends.

As far as RMS-X residuals are concerned, this method implies a previous handling of the whole spectra with different combinations of mathematical treatments (MSC, SNV, DT or SNV-DT), derivatives, and smoothing procedures. After optimization the highest percentage of correctly classified samples was obtained by applying the Detrend (2,4,4,1) combination which was also the pre-treatment selected for heavy metal calibration. The results are shown in Table 5; a better performance was obtained by using RMS-X residuals than SIMCA for both analytical approaches, pure tea vs. blends, and the discrimination of the percentage of blending for each tea type.

As far as the classification of the teas according to the purity of the samples is concerned, 100% of the pure samples were correctly classified in calibration and only for pure red tea the classification was successful for 91% of the samples in validation. To consider the different blends, the percentage of correctly classified samples was between 93% for black blends and 98% for red blends in calibration and between 71% and 97% for black and green blends respectively. The high sensitivity and in particular the high specificity of the RMS-X residuals method is noteworthy in both the calibration and the validation sets, which means that only the samples belonging to a certain group are included in this group.

Finally, the classification according to the percentage of blending for each type of tea showed very good results for the classification stage with values ranging from 70% for blends containing between 75% and 50% of black tea to 100%. However, although the percentage of samples correctly classified in the validation set was also 100% for the blends containing >95% of each tea, the values were much lower for the remainder of the blends and even reached 0% for the 75–85% blends. One of the reasons is that there were 12 categories and the number of samples in some categories were extremely low. Despite this, the specificity of the method was again very high (0.87–1).

These results agree with those of previous studies which showed the feasibility of discriminating teas by using NIR technology. The treatment of NIR spectra with different chemometric tools has therefore allowed the successful discrimination of teas according to their quality [29,59], variety [68,69], degree of fermentation [94], geographical origin [15], and blending [41]. On the other hand, RMS-X residuals showed a satisfactory level of discrimination which is in agreement with previous studies which pointed out the feasibility of using this method to distinguish the samples according to their origin [95,96] and even according to the percentage of the mixture and variety [80]. Indeed, this method has shown better performance than other classification methods such as SIMCA as has previously been reported by Revilla et al. [97].

## 4. Conclusions

The results allowed us to conclude that it was possible to discriminate pure tea samples according to their content in heavy metals (As, Pb, Cu, Al, Hg). Red teas were therefore characterized by their higher content of As and Pb, green teas were the only ones containing Hg, and black teas showed the highest levels of copper. 

Moreover, NIRS technology has been shown to be a suitable technique for determining the heavy metal composition of tea leaves. It was possible to predict all of the heavy metals analyzed with high RSQ (>0.89) and RPD (>3) values. The range of applicability of the equations is comparable to the reference method, and no significant differences between the reference and the predicted values were observed. Finally, NIR spectroscopy together with the RMS-X residual method allowed the discrimination of pure tea samples (black, red and green) and their blends with an accuracy of 98.3% in calibration while 92.3% of the total samples were correctly classified in validation. When the samples were discriminated according to the percentage of blending, 100% of the samples of 10 out of 12 groups were correctly classified in calibration although only the groups with a level of pure tea of >95% showed 100% of the samples to be correctly classified at the validation stage. NIR spectroscopy has therefore been shown to be a suitable technique for detecting the presence of other tea types in pure samples and for quantifying the heavy metal content in a multiparametric, fast, and simple way without prior treatment of the sample.

## Figures and Tables

**Table 1:** Total contents of heavy metal in pure black, green and red tea. Mean value and standard deviation (SD).

	N	Al (g/kg)	Pb (mg/kg)	As (mg/kg)	Hg (mg/kg)	Cu (mg/kg)
Black tea	7	2.04 ± 0.23 ^a^	0.56 ± 0.28 ^a^	0.04 ± 0.01 ^a^	0.00 ± 0.00 ^a^	17.31 ± 2.60 ^b^
Red tea	8	2.36 ± 0.25 ^b^	1.21 ± 0.36 ^b^	0.19 ± 0.09 ^b^	0.00 ± 0.00 ^a^	19.87 ± 2.78 ^b^
Green tea	11	2.52 ± 0.29 ^b^	0.98 ± 0.40 ^b^	0.09 ± 0.02 ^a^	0.01 ± 0.01 ^b^	12.19 ± 2.40 ^a^

^a,b^ Different letters in the same column indicate statistically significant differences; analysis of variance (ANOVA); post hoc: Tukey HSD test; *p* < 0.05.

**Table 2:** Mineral composition of the calibration and validation sets.

	Calibration Set (n = 257)				External Validation Set (n = 65)			
Mineral	Minimun	Maximun	Mean	SD	Minimun	Maximun	Mean	SD
Al (g/kg)	1.8490	3.0470	2.3705	0.270	1.9540	2.9524	2.3153	0.223
Pb (mg/kg)	0.2810	1.7100	0.8943	0.333	0.3170	1.4952	0.9003	0.293
As (mg/kg)	0.0291	0.3910	0.1101	0.049	0.0305	0.2263	0.1099	0.048
Hg (mg/kg)	0.0006	0.0189	0.0096	0.004	0.0022	0.0185	0.0096	0.004
Cu (mg/kg)	8.4400	23.9140	15.9984	3.641	8.9430	23.7200	15.4139	3.728

**Table 3:** Statistical descriptors for the best calibration equations obtained from NIR spectra and mineral composition data obtained by ICP-MS on tea samples.

Mineral	N	Mean	SD	Min Est.	Max Est.	SEC	SECV	RSQ	RPD
Al (g/kg)	239	2.3537	0.2565	1.584	3.123	0.084	0.109	0.893	3.11
Pb (mg/kg)	243	0.8784	0.3285	0.000	1.864	0.070	0.091	0.955	4.71
As (mg/kg)	235	0.1051	0.0444	0.000	0.238	0.012	0.018	0.923	3.62
Hg (mg/kg)	244	0.0066	0.0057	0.000	0.024	0.001	0.002	0.966	5.17
Cu (mg/kg)	243	16.0964	3.6119	5.261	26.932	0.625	0.850	0.970	5.78

N: number of samples after removing the outliers; SD: standard deviation; SEC: standard error of calibration; SECV: standard error of cross validation; RSQ: multiple correlation coefficient; RPD: ratio performance deviation.

**Table 4:** External validation for the NIR determination of minerals in different types of tea.

Mineral	*p* (Level of Significance)	RMSE
Al	0.890	0.112
Pb	0.792	1.003
As	0.778	0.182
Hg	0.684	0.027
Cu	0.796	0.021

**Table 5:** Results of the classification of the pure teas vs. tea blends and of the classification by percentage of blending by SIMCA and by RMSX-residuals method for the best mathematical pre-treatment applied to the NIR spectra.

	SIMCA Method		RMSX-Residuals Detrend (2,4,4,1)					
	**% Samples Correctly Classified**		**% Samples Correctly Classified**		**Sensitivity**		**Specificity**	
**Component**	**Calibration**	**Validation**	**Calibration**	**Validation**	**Calibration**	**Validation**	**Calibration**	**Validation**
Pure tea vs. blends								
Black pure	100.00	100.00	100.00	100.00	1.00	1.00	1.00	1.00
Red pure	98.46	93.75	100.00	90.91	1.00	1.00	1.00	1.00
Green pure	45.56	10.00	100.00	100.00	1.00	0.91	1.00	1.00
Black blends	0.00	0.00	93.33	71.43	0.93	0.71	0.99	1.00
Red blends	24.05	25.00	98.75	94.74	0.99	0.95	0.99	0.97
Green blends	77.44	75.00	97.79	96.97	0.98	0.97	1.00	0.91
Percentage of blending								
>95% black	100.00	100.00	100.00	100.00	1.00	1.00	1.00	0.99
95–85% black	0.00	0.00	100.00	50.00	1.00	0.50	1.00	1.00
85–75% black	0.00	0.00	100.00	0.00	1.00	0.00	1.00	1.00
75–50% black	0.00	0.00	70.00	33.33	0.70	0.33	1.00	0.99
>95% red	91.67	88.24	100.00	100.00	1.00	1.00	1.00	0.95
95–85% red	0.00	0.00	100.00	20.00	1.00	0.20	1.00	1.00
85–75% red	0.00	0.00	100.00	0.00	1.00	0.00	1.00	1.00
75–50% red	0.00	0.00	100.00	66.67	1.00	0.97	0.99	0.92
>95% green	100.00	100.00	100.00	100.00	1.00	1.00	1.00	1.00
95–85% green	0.00	0.00	100.00	62.50	1.00	0.97	1.00	1.00
85–75% green	0.00	0.00	100.00	0.00	1.00	0.96	1.00	0.99
75–50% green	0.00	0.00	98.55	88.24	1.00	0.97	1.00	0.87

Black blends: blends with more than 50% of black tea with red or green tea. Red blends: blends with more than 50% of red tea with black or green tea. Green blends: blends with more than 50% of green tea with black or red tea.

## Data Availability

The data presented in this study are available on request from the corresponding author. The data are not publicly available due to privacy.
